# *Clostridioides difficile* Activates Human Mucosal-Associated Invariant T Cells

**DOI:** 10.3389/fmicb.2018.02532

**Published:** 2018-10-25

**Authors:** Isabel Bernal, Julia Danielle Hofmann, Björn Bulitta, Frank Klawonn, Annika-Marisa Michel, Dieter Jahn, Meina Neumann-Schaal, Dunja Bruder, Lothar Jänsch

**Affiliations:** ^1^Institute of Medical Microbiology and Hospital Hygiene, Infection Immunology, Otto von Guericke University Magdeburg, Magdeburg, Germany; ^2^Structure and Function of Proteins, Helmholtz Centre for Infection Research, Braunschweig, Germany; ^3^ESF Graduate School ABINEP, Magdeburg, Germany; ^4^Department of Bioinformatics and Biochemistry, Braunschweig Integrated Centre of Systems Biology, Technical University of Braunschweig, Braunschweig, Germany; ^5^Department of Computer Science, Ostfalia University of Applied Sciences, Wolfenbüttel, Germany; ^6^Department of Microbiology, Braunschweig Integrated Centre of Systems Biology, Technical University of Braunschweig, Braunschweig, Germany; ^7^Leibniz Institute DSMZ - German Collection of Microorganisms and Cell Cultures, Braunschweig, Germany; ^8^Immune Regulation, Helmholtz Centre for Infection Research, Braunschweig, Germany

**Keywords:** *C. difficile* infection, MAIT cells, MR1-antigen presentation, riboflavin synthesis, mucosal immunity

## Abstract

*Clostridioides difficile* infection (CDI) causes severe inflammatory responses at the intestinal mucosa but the immunological mechanisms underlying CDI-related immunopathology are still incompletely characterized. Here we identified for the first time that both, non-toxigenic strains as well as the hypervirulent ribotypes RT027 and RT023 of *Clostridioides difficile* (formerly *Clostridium difficile*), induced an effector phenotype in mucosal-associated invariant T (MAIT) cells. MAIT cells can directly respond to bacterial infections by recognizing MR1-presented metabolites derived from the riboflavin synthesis pathway constituting a novel class of antigens. We confirmed functional riboflavin synthesis of *C. difficile* and found fixed bacteria capable of activating primary human MAIT cells in a dose-dependent manner. *C. difficile*-activated MAIT cells showed an increased and MR1-dependent expression of CD69, proinflammatory IFNγ, and the lytic granule components granzyme B and perforin. Effector protein expression was accompanied by the release of lytic granules, which, in contrast to other effector functions, was mainly induced by IL-12 and IL-18. Notably, this study revealed hypervirulent *C. difficile* strains to be most competent in provoking MAIT cell responses suggesting MAIT cell activation to be instrumental for the immunopathology observed in *C. difficile*-associated colitis. In conclusion, we provide first evidence for a link between *C. difficile* metabolism and innate T cell-mediated immunity in humans.

## Introduction

*Clostridioides difficile* (formerly *Clostridium difficile*) is a Gram-positive, anaerobic bacterium causing severe and recurrent colitis with a high mortality rate in humans (Kwon et al., 2015; Lawson et al., 2016). Due to its spore-forming capacity *C. difficile* is highly resistant to clinical hygiene measures (Dubberke et al., 2007), which represents a major drawback for preventing pathogen spreading in health care institutions. In fact, nosocomial *C. difficile-*associated colitis (CDAC) has reached highest medical and economical relevance in Germany, with increasing incidence (Bauer et al., 2011) and with an economic burden of up to €464 million/year for the German health-care system (Reigadas Ramírez and Bouza, 2018).

*Clostridioides difficile* strains are discriminated as ribotypes (RTs) and toxinotypes based on information from rRNA-based phylogenetic analyses and characteristics of the pathogenicity locus where enterotoxin and cytotoxin genes, *tcdA* and *tcdB*, are arranged (Duerden et al., 2001). *C. difficile* strains expressing neither toxin A (TcdA) nor toxin B (TcdB) are defined as non-toxigenic. Strains of ribotype RT084 are prototypic non-toxigenic strains, which are prevalent in symptomatic patients in sub-Saharan Africa (Janssen et al., 2016). A TcdA/B-toxigenic *C. difficile* strain with RT012 was the first fully sequenced and annotated *C. difficile* strain and its genome still serves as reference (Sebaihia et al., 2006). The so-called hypervirulent *C. difficile* strains with RT027 or RT023 produce, in addition to TcdA and TcdB, the binary toxin, also known as *C. difficile* transferase (CDT) (Duerden et al., 2001). *C. difficile* strain with RT027 caused large epidemics across the developed world with substantial morbidity and mortality (Kuijper et al., 2008; He et al., 2013). In Sweden, strains with RT023 were identified as the causative agent of recurrent CDI (He et al., 2013). Although toxin-associated pathogenicity is well studied, the understanding of the often destructive immunological processes involved in human CDI remain rudimentary (Pothoulakis, 1996, 2000; Chandrasekaran and Lacy, 2017).

The recently identified mucosal-associated invariant T (MAIT) cells represent an innate-like T cell subset with antibacterial properties that is highly abundant in the human blood and especially at mucosal surfaces. In the intestinal lamina propria they constitute up to 10% of total T cells (Treiner et al., 2003). MAIT cells express high levels of the C-type lectin CD161 and the T cell receptor (TCR) α-chain Vα7.2 (Tilloy et al., 1999). This semi-invariant TCR, together with a limited TCRβ repertoire, restricts them to the major histocompatibility complex (MHC) class I-related protein MR1, which is expressed on the surface of antigen presenting cells and epithelial cells (Le Bourhis et al., 2010; Dusseaux et al., 2011; Moreira et al., 2017). MR1 presents small molecular ligands derived from bacterial riboflavin (vitamin B2) precursor 5-amino-6-d-ribitylaminouracil (5-A-RU) (Kjer-Nielsen et al., 2012; Corbett et al., 2014), thereby constituting a new antigen class for innate-like T cell activation. Their antigen specificity and their effector memory-like phenotype defines the innate-like phenotype of MAIT cells and enables them to immediately execute effector functions upon stimulation (Dusseaux et al., 2011). Beside the semi-invariant TCR, MAIT cells also show high constitutive expression of the IL-12 and IL-18 receptors (Le Bourhis et al., 2010; Slichter et al., 2016) rendering them sensitive for cytokine-mediated activation. TCR-activated MAIT cells can mediate cytotoxicity by lytic granules containing effector molecules such as perforin and a set of granzymes. In previous studies, we have characterized the molecular effector inventory of unstimulated human MAIT cells revealing high expression levels of granzyme A, K, and M (Bulitta et al., 2018). In contrast, granzyme B expression is only induced upon MAIT cell activation (Kurioka et al., 2015). In addition, the expression of immune-modulating Th1- and Th17-related cytokines such as IFNγ and IL-17 are inducible as well in MAIT cells upon activation (Dusseaux et al., 2011; Le Bourhis et al., 2013). Thus, MAIT cells on the one hand can exert cell-contact dependent anti-bacterial cytotoxicity, while at the same time they are considered as systemic boosters of inflammation with in part detrimental effects in certain disease settings, such as multiple sclerosis (Willing et al., 2014). All so far described human MAIT cell activating bacteria, including *E. coli, S. aureus, M. tuberculosis*, and *S. pneumoniae*, exhibit a functional riboflavin synthesis pathway (Dias et al., 2016; Jiang et al., 2016; Johansson et al., 2016; Kurioka et al., 2018), whereby *E. coli* constitutively produces riboflavin (Vitreschak et al., 2002). While genomic data suggest the existence of a functional riboflavin pathway also in *C. difficile* (Janoir et al., 2013) experimental evidence of functional gene expression and riboflavin synthesis as well as MAIT cell-activating potential by *C. difficile* is still lacking. Here, we studied the responsiveness of peripheral human MAIT cells and identified a MAIT cell effector phenotype induced by *C. difficile* suggesting their potential role in the immunopathology of CDAC.

## Materials and Methods

### *C. difficile* Cultures

*Clostridioides difficile* clinical isolates were provided by Leibniz Institute DSMZ – German Collection of Microorganisms and Cell Cultures (Braunschweig). DSM 28196 (RT027), DSM 28666 (RT084), DSM 29745 (RT001) (depositor Uwe Groß), DSM 28645 (RT012) (depositor Ralf Gerhard), DSM 102859 (RT023) (depositor: Lutz von Müller) strains were cultured in riboflavin-free casamino acids containing medium (CDMM) under anaerobic conditions (Neumann-Schaal et al., 2015; Riedel et al., 2017). Cells were harvested at the mid exponential phase (1/2 OD_max_). Bacterial numbers were determined using a Neubauer improved counting chamber (C-Chip, NanoEnTek). Bacterial cell pellets were harvested by centrifugation (13.000 g, 10 min, 4°C) and fixed with 2% paraformaldehyde (PFA) solution, were washed three times with PBS and stored at °80°C. Prior PBMC stimulation, the bacterial cells were resuspended in PBS to a final concentration of 3 × 10^8^ bacteria/ml.

### RT-PCR

Bacterial RNA was isolated using Qiagen RNeasy Mini Kit (Qiagen) according to manufacturer’s instructions. RT-PCR for *ribD* and *ribE* gene was performed using Verso 1-Step RT-PCR Hot-Start kit (Thermo Fisher Scientific). *RibD* encodes for riboflavin biosynthesis bifunctional diamino-hydroxy-phospho-ribosyl-amino-pyrimidine deaminase/5-amino-6-(5-phosphoribosylamino) uracil reductase, which generates phosphorylated 5-A-RU, the precursor of the MR1-binding ligand 5-A-RU. 5-A-RU is converted by the *ribE* gene product lumazine synthase into 6,7-dimethyl-8-ribityllumazine (RL-6,7-diMe), which then can be processed to riboflavin. Sequences for *ribD* primers are, 5′ end to 3′ end, forward AATCAGTAAGTCTAGATGG, reverse CTGTCATTGAGAGTAGCACC, for *ribE* primers forward CAGCCGATGTTATGATGGAG, and reverse CTCCAACATTCTTTGTCAAGAG.

### Blood Donations

This study was conducted in accordance with the rules of the Regional Ethics Committee of Lower Saxony, Germany and the declaration of Helsinki. Buffy coats from blood donations of healthy human volunteers, who provided informed consent, were obtained from the Institute for Clinical Transfusion Medicine, Klinikum Braunschweig, Germany. Blood donors’ health was assessed prior blood donation. This procedure also includes standardized laboratory tests for infections with HIV1/2, HBV, HCV, and *Treponema pallidum* (serology and/or nucleic acid testing) and hematological cell counts.

### PBMC Isolation and Stimulation

Buffy Coats were produced from whole blood donations by using the Top & Bottom Extraction Bag System (Polymed Medical Devices). Peripheral blood mononuclear cells (PBMCs) were isolated from buffy coats by Ficoll^®^ Paque PLUS density gradient centrifugation (GE Healthcare GmbH). PBMCs were rested overnight in RPMI 1640 medium (Gibco/Life Technologies) supplemented with 10% fetal bovine serum gold (PAA Laboratories), 2 mM L-glutamine, 50 units/ml penicillin and 50 μg/ml streptomycin (all Gibco/Life Technologies) at 37°C in a humid 7.5% CO_2_ atmosphere. 0.5 × 10^6^ PBMCs were either left untreated or stimulated with PFA-fixed bacteria at different multiplicities of infection (MOI; bacteria per PBMC cell) for 6, 12, 17, and 20 h at 37°C. If indicated, PBMCs were treated with blocking antibodies against MR1 (26.5, BioLegend, 20 μg/ml) and/or against IL-12 p35 (clone B-T21, eBioscience, 5 μg/ml) or IL-18 (clone 126-2H, MBL International, 1:200 dilution) 1 h prior stimulation with PFA-fixed bacteria.

### Antibodies

For cytometric assessment of MAIT cell phenotype, bulk PBMCs were stained with LIVE/DEAD^TM^ Fixable Blue Dead Cell Stain Kit (Invitrogen) and Fc receptor blocking reagent (Miltenyi Biotec) and a combination of the following antibodies (from BioLegend except as noted): CD3 BV655 (clone OKT3), CD8 BV711 (clone RPA-T8), CD161 APC (clone DX12, BD Biosciences), Vα7.2 (clone 3C10), CD69 PE (clone FN50), CD107a PerCP-Cy5.5 (clone H4A3), granzyme B Pacific Blue (clone GB11), perforin FITC (clone), and interferon γ APC-Cy7 (clone 4S.B3).

### Extracellular and Intracellular MAIT Cell Staining

Stimulated PBMCs were washed with PBS and stained with Fc receptor blocking reagent (Miltenyi Biotec) and LIVE/DEAD^TM^ Fixable Blue Dead Cell Stain Kit (Invitrogen). Cells were washed with FACS buffer and stained for extracellular surface marker. Cell fixation and permeabilization were performed using BD Cytofix/Cytoperm^TM^ (BD Biosciences) following intracellular staining for cytokines and cytolytic molecules. Cells were washed twice with permeabilization buffer and cell pellet was resuspended in FACS buffer and subsequently analyzed on BD LSR-II SORP and BD LSR-Fortessa flow cytometer. Data analysis was then carried out by FlowJo (TreeStar, v10.4.2) and Prism (GraphPad Software, v7.0c). To determine significant differences, Wilcoxon matched-pairs signed rank test was used.

### Analysis of Bacterial Riboflavin

For the determination of the riboflavin content 30 ml culture were harvested anaerobically in the exponential phase as described previously (Dannheim et al., 2017). Cell samples were centrifuged (10 min, 10.000 g, 4°C), washed with sterile, anaerobe PBS and the precipitated cells were immediately frozen in liquid nitrogen. Cell samples were resuspended in 100 μL 1 M NaOH, vigorously mixed (5 min, 2000 rpm), neutralized with 400 μL 1 M potassium phosphate buffer pH 6.0 and centrifuged (5 min, 17.000 g, 4°C). The supernatant was sterile filtered and the riboflavin concentration was determined with a 1260 Infinity HPLC system equipped with a fluorescence detector (Agilent Technologies) and a Poroshell 120 EC – C18 separation column (3.0 mm × 50 mm, particle size 2.7 mm, Agilent Technologies). The samples were measured at 35°C with a flow rate of 1 ml min-1 according to Glinko et al. (2008), using a programmed gradient mobile phase A (25 mM NaH2PO4, pH 2.5) and mobile phase B (methanol) with the following modifications: 0–1 min, linear gradient from 1 to 12% B; 1–1.03 min, step from 12 to 30% B; 1.03–10 min, isocratic at 30% B; 10–15 min, linear gradient from 30 to 100% B; 15–17 min, isocratic at 100% B; 17–17.03 min, step from 100 to 1% B; 17.03–20 min, isocratic at 1% B (column equilibration). Riboflavin was detected by fluorescence detection (FLD) at 450 nm excitation and 530 nm emission according to Chen et al. (2009).

### Quantification of Cytokines

0.5 × 10^6^ PBMCs were either left untreated or stimulated with *C. difficile* ribotypes RT084, RT012, RT001, RT023, and RT027 at MOI 1 for 20 h at 37°C in duplicates. Supernatants of respective samples were pooled and enzyme-linked immunosorbent assays were performed to detect human IL-12 using Human IL-12 (p70) ELISA MAX^TM^ Standard (BioLegend) and human IL-18 using IL-18 Human ELISA Kit (Invitrogen).

## Results

### *C. difficile* Induces Activation and Effector Functions in Primary Human MAIT Cells

Genome analysis of *C. difficile* indicated the presence of a functional riboflavin pathway and thus suggested their ability to activate MAIT cells. However, expression of riboflavin pathway genes has not been experimentally proven so far. Therefore, we first validated the expression of riboflavin biosynthetic enzymes in two *C. difficile* strains grown in riboflavin-free medium: *C. difficile* strain with RT084 represents a prevalent non-toxigenic strain in hospitalized and non-hospitalized patients and RT023 represents a TcdA/B/CDT-toxigenic, hypervirulent strain causing diarrhea with high mortality rate in hospitalized patients. Gene expressions were analyzed for *ribD* that generates the MR1-binding ligand precursor 5-A-RU and additionally for *ribE* that can influence 5-A-RU level according to the KEGG pathway database as well. Indeed, we observed substantial *ribD* and *ribE* expression on mRNA level in both clinical isolates (Supplementary Figure 1), indicating an active riboflavin biosynthesis pathway in proliferating *C. difficile.*

**FIGURE 1:**
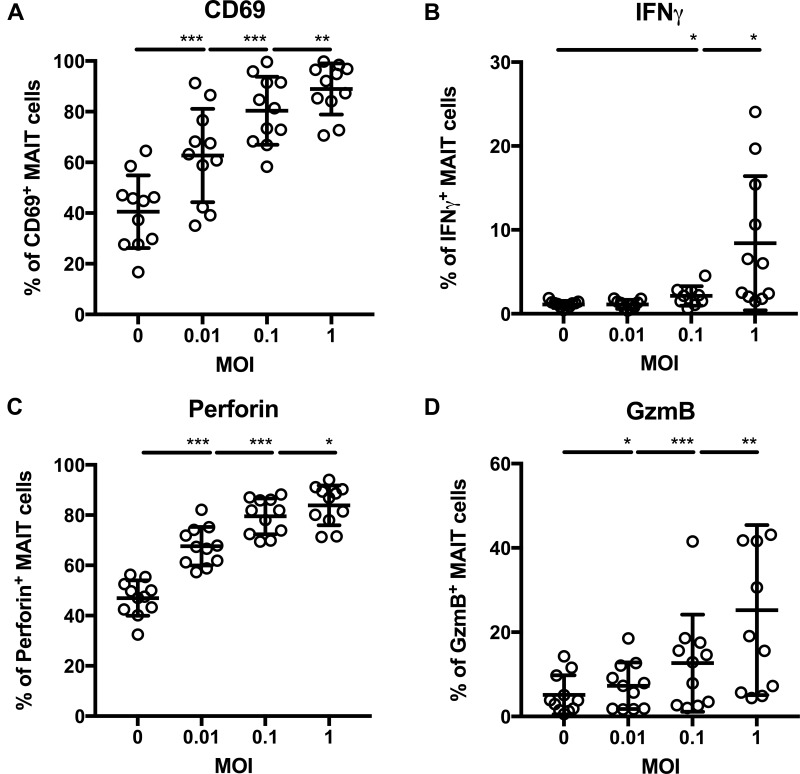
Dose-dependent activation of primary human MAIT cells following stimulation with *Clostridioides difficile* (ribotype RT023). PBMCs were isolated from healthy donors and stimulated overnight with paraformaldehyde-fixed *C. difficile* isolate ribotype RT023 followed by flow cytometric analyses of surface staining of CD69 **(A**) and intracellular staining of IFNγ **(B)**, perforin **(C)**, and granzyme B [GzmB **(D)**]. Mean percentages ± SD are shown. Cells were gated on CD161^++^Va7.2^+^CD3^+^ T cells (MAIT cells). Wilcoxon signed rank test for paired samples was used to detect significant differences and determine *p*-values (^∗^*p* < 0.05, ^∗∗^*p* < 0.01, and ^∗∗∗^*p* < 0.001). Combined data from three independent experiments and 9–11 donors are shown.

We next examined whether *C. difficile* is able to activate MAIT cells *in vitro*. To this end, we selected a hypervirulent strain with RT023 and probed its ability to activate MAIT cells from healthy individuals (gating strategy depicted in Supplementary Figure 2). As established for other bacterial species before (Dias et al., 2016), MAIT cell activation was tested by using PFA-fixed non-viable *C. difficile* that were applied to PBMCs from different donors at different MOIs (0.01, 0.1, and 1) for 20 h. Indeed, *C. difficile* RT023 caused notable MAIT cell activation already at low bacterial doses (MOI 0.01) as indicated by the significant surface expression of CD69, while 10-times higher bacterial numbers were required to induce significant IFNγ expression (Figures 1A,B). Interestingly, as for CD69 induction, MOI of 0.01 was validated to be sufficient to trigger the cytotoxic effector phenotype of MAIT cells, which is characterized by significantly increased expression of the major lytic granule components perforin and granzyme B (GzmB) (Figures 1C,D). This indicates that MAIT cell cytotoxicity and proinflammatory cytokine response have clearly distinct antigenic activation thresholds in CDI. In conclusion, *C. difficile* is competent to generate MAIT cell-activating ligands and to induce MAIT cell effector responses.

### *C. difficile* Induces MAIT Cell Activation and Effector Function via MR1 or IL-12/IL-18

**FIGURE 2:**
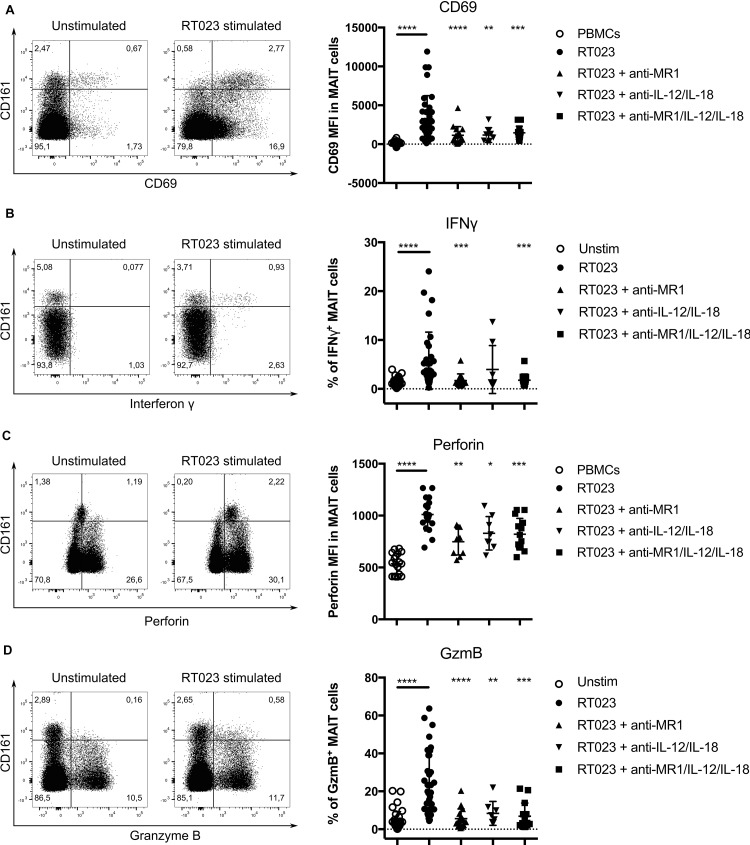
MR1- and cytokine-dependent activation of primary human MAIT cells following stimulation with *Clostridioides difficile* (ribotype RT023). PBMCs were isolated from healthy donors [**(A,B,D**) *n* = 8–36, for **(C)** *n* = 8–14] and stimulated with *C. difficile* clinical isolate with ribotype RT023 at MOI 1 for 20 h followed by flow cytometric analyses of indicated parameters. Mean percentages ± SD are shown. Cells were gated on CD161^++^Va7.2^+^CD3^+^ T cells (MAIT cells). Wilcoxon signed rank test for paired samples was used to detect significant differences and determine *p*-values (^∗^*p* < 0.05, ^∗∗^*p* < 0.01, ^∗∗∗^*p* < 0.001, and ^∗∗∗∗^*p* < 0.0001). Left: representative plots of CD3^+^ T cells. Right: combined data from 12 independent experiments and 8–36 donors are shown.

Mucosal-associated invariant T cell activation can occur either MR1-dependently or co-dependent on both MR1 and the cytokines IL-12 and IL-18 (Le Bourhis et al., 2010). To dissect the importance of these two stimulation conditions for *C. difficile*-mediated MAIT cell activation we stimulated PBMCs with the hypervirulent *C. difficile* strain (RT023) in the presence of MR1 and/or IL-12/IL-18 blocking antibodies and analyzed MAIT cell responses after 20 h. MAIT cells from individual donors were stimulated by using a MOI of 1 that, in the absence of blocking antibodies, is able to cause robust and significant MAIT cell responses including IFNγ (Figure 1). Since IFNγ and GzmB expression is upregulated in activated MAIT cells, we measured the frequency of MAIT cells positive for these markers. For CD69 and perforin, which both showed basal expression levels already in unstimulated MAIT cells, changes in median of mean fluorescence intensity (MFI) were determined (Figures 2A,C). As shown before (Figure 1), MAIT cells readily responded to *C. difficile* stimulation by significant upregulation of CD69, IFNγ, perforin and GzmB (Figures 2A–D). CD69 induction could significantly (but not completely) be blocked by anti-MR1, anti-IL-12/IL-18 and a combination of both, suggesting that *C. difficile*-induced CD69 expression is MR1- and cytokine-dependent (Figure 2A). Interestingly, IFNγ expression could be blocked almost completely by anti-MR1. However, in contrast to CD69, perforin and GzmB, blockade of IL-12/IL-18 signaling alone did not significantly affect IFNγ production in *C. difficile*-stimulated MAIT cells (Figure 2B). To determine whether IL-12/IL-18 has an effect on IFNγ response of MAIT cells, we stimulated PBMCs with RT023 together with recombinant IL-12/IL-18 resulting in a pronounced IFNγ expression (Supplementary Figure 3). Regarding perforin, the blockade of MR1 or/and IL-12/IL-18 signaling resulted in a significant reduction of its expression (Figure 2C). However, combined application of MR1 and IL-12/IL-18 blocking antibodies was not sufficient to completely inhibit perforin expression in *C. difficile*-stimulated MAIT cells, indicating that its expression is regulated also by other pathways. The induction of GzmB expression following stimulation by *C. difficile* was almost completely blocked by anti-MR1 and significantly reduced by anti-IL-12/IL-18 as well as the combination of both treatments. In conclusion, despite obvious donor-specific variations in the expression levels of the analyzed markers, we show that *C. difficile* activates effector functions in human MAIT cells in a MR1- and in part cytokine-dependent manner.

**FIGURE 3:**
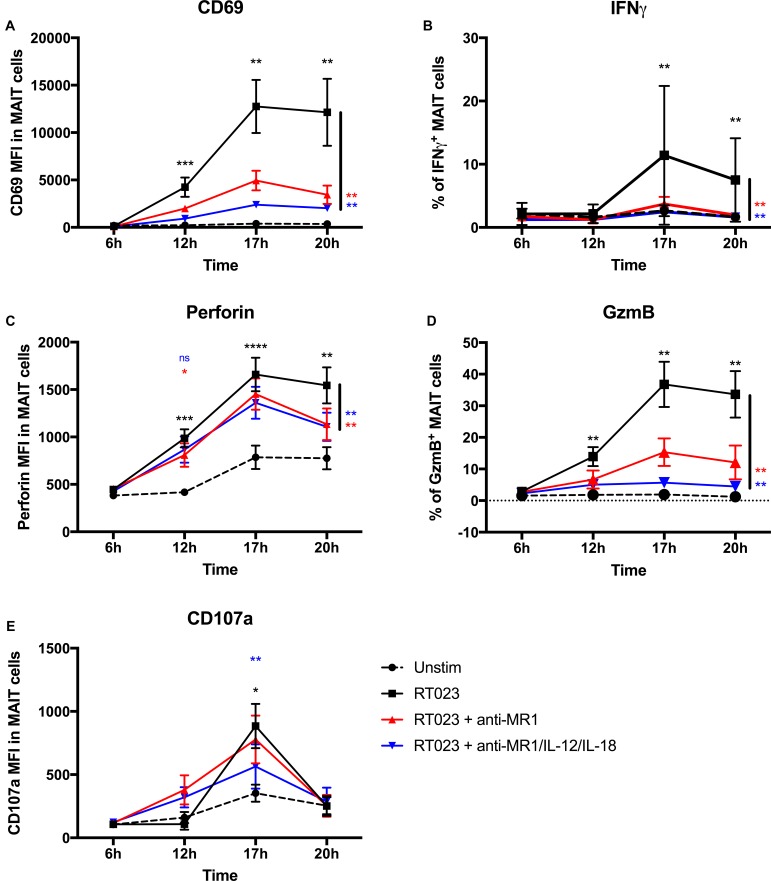
Kinetics of parameter expression of primary human MAIT cells following stimulation with *Clostridioides difficile* (ribotype RT023). PBMCs were isolated from healthy donors and stimulated with clinical *C. difficile* isolate with ribotype RT023 at MOI 1 for indicated time followed by flow cytometric analyses of surface staining of CD69 **(A)**, CD107a **(E)** and internal staining of IFNγ **(B)**, perforin **(C)**, and granzyme B [GzmB **(D)**]. Means ± SEM are shown. Cells were gated on CD161^++^Va7.2^+^CD3^+^ T cells (MAIT cells). Black asterisks indicate significant differences of marker expression in stimulated MAIT cells compared to the unstimulated controls. Blue and red asterisks indicate significant differences of marker expression in stimulated MAIT cells compared to antibody treated samples. 8–12 donors per group were tested, by Wilcoxon signed rank test for paired samples and determine *p*-values (^∗^*p* < 0.05, ^∗∗^*p* < 0.01, ^∗∗∗^*p* < 0.001, and ^∗∗∗∗^*p* < 0.0001). Combined data from five independent experiments are shown.

Next, we probed the kinetics of *C. difficile*-induced MAIT cell responses by investigating CD69, IFNγ, perforin, and GzmB expression in response to *C. difficile* strain with RT023 at 6, 12, 17, and 20 h (Figure 3). Additionally, we examined the surface level of CD107a to monitor potential degranulation of lytic granules. We observed significant upregulation of CD69, perforin, and GzmB compared to unstimulated cells at 12 h with a peak at 17 h post *C. difficile* stimulation (Figures 3A,C,D). At 17 h post stimulation, we also detected the highest increase in IFNγ expression, which, however, showed relatively strong donor variation (Figure 3B). Interestingly, at 17 h we observed lytic granule degranulation indicated by the surface expression of CD107a and therefore cytotoxic activity of MAIT cells in response to *C. difficile* (Figure 3E). In general, time course experiments confirmed CD69, IFNγ, perforin, and GzmB expression to be dependent on MR1 as described before for the 20 h stimulation experiments (Figure 2), and an additional contribution of IL-12/IL-18 became apparent for CD69 and GzmB (Figures 3A,D). In contrast, IFNγ and perforin induction were found to be mainly MR1-dependent at all investigated time points, since additional blockade of IL-12/IL-18 showed only limited effects. In conclusion, *C. difficile*-induced MAIT cell response was detectable earliest at 12 h following stimulation with a peak response at 17 h. Expression dynamics of the effector molecules IFNγ, GzmB and perforin were found largely MR1-dependent while MAIT cell degranulation appears to involve additional IL-12/IL-18 cytokine signaling.

### Hypervirulent *C. difficile* Strains Provoke Strongest MAIT Cell Activation and Effector Function

**FIGURE 4:**
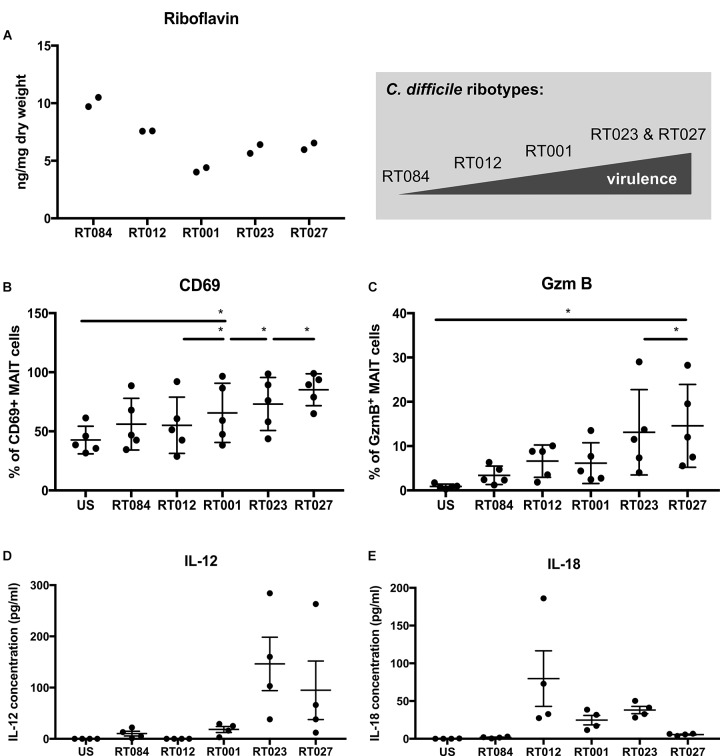
MAIT cell activation by *Clostridioides difficile* clinical isolates that differ in riboflavin metabolism. Riboflavin content of *C. difficile* clinical isolates was measured by fluorescence detection and ng/mg dry weight ± SD is shown **(A)**. Data of technical replicates from two cultivations are shown. PBMCs were isolated from healthy donors and stimulated with *C. difficile* clinical isolates of the ribotypes RT084, RT012, RT001, RT023, and RT027 at MOI1 for 20 h. **(B)** Followed by flow cytometric analyses of activation marker CD69 **(B)** and intracellular GzmB **(C)**. Means ± SD are shown. Cells were gated on CD161^++^Va7.2^+^CD3^+^ T cells (MAIT cells). Wilcoxon signed rank test for paired samples was used to detect significant differences and determine *p*-values (^∗^*p* < 0.05). Combined data from two independent experiments using five donors are shown. IL-12 **(D)** and IL-18 **(E)** were quantified in PBMC supernatant. Means ± SD are shown. Data from one experiment and four donors are shown.

Next to their antibacterial function MAIT cell activation can result in exaggerated release of proinflammatory mediators and may thus contribute to excessive immunopathology at the site of infection (Shaler et al., 2017). Since the toxins of the hypervirulent ribotypes are considered to be the causative agents for detrimental courses of CDAC, we tested whether these strains would be particularly active in riboflavin synthesis and whether this might be the basis for an extraordinary strong MAIT cell activation. To this end, we quantified riboflavin level by fluorescence detection in five different *C. difficile* clinical isolates. These included a non-toxigenic strain (ribotype RT084), two TcdA/B-toxigenic strains (ribotypes RT012 and RT001) as well as two hypervirulent TcdA/B/CDT-toxigenic strains (ribotypes RT023 and RT027). Since it is known, that riboflavin biosynthesis is highly regulated in *C. difficile* (Hofmann et al., 2018), we have harvested and fixed bacteria from the same culture to analyze MAIT cell response. Indeed, we observed strain-dependent differences with respect to riboflavin level (Figure 4A). Surprisingly, highest riboflavin content was not detected in the hypervirulent strains (ribotypes RT023 and RT027) but in the non-toxigenic strain. Fractions of the bacterial cultures used to determine the riboflavin content were simultaneously used to determine their MAIT cell-activating capacity. In particular, we wondered whether the riboflavin level would correlate with the magnitude of MAIT cell responses and thus would serve as an indicator for the contribution of MAIT cells to the inflammatory response during CDAC. Interestingly, despite containing highest riboflavin level, the non-toxigenic strain (ribotype RT084) exhibits the least capacity to induce MAIT cell activation (CD69) and cytotoxic effector function (GzmB) (Figures 4B,C). Strikingly, strongest CD69 and GzmB induction was observed in MAIT cells stimulated with the hypervirulent *C. difficile* isolates (ribotype RT023 and RT027) that only contained intermediate riboflavin levels.

Finally, we asked the question whether hypervirulent *C. difficile* clinical isolates (ribotype RT023 and RT027) evoke different cytokine pattern in antigen presenting cells. To answer this, we determined the IL-12 and IL-18 concentrations in the supernatant of *C. difficile*-stimulated PBMCs, revealing that hypervirulent *C. difficile* isolates (ribotype RT023 and RT027) induce a higher IL-12 secretion by antigen presenting cells than non-hypervirulent strains (Figure 4D). However, IL-18 secretion was only moderate in hypervirulent strains (Figure 4E), suggesting that hypervirulent strains induce an IL-12 specific cytokine pattern in antigen presenting cells.

In conclusion, we detected superior MAIT cell responses triggered by hypervirulent *C. difficile* isolates, suggesting a role of MAIT cells in inflammation and immunopathology in CDAC.

## Discussion

*Clostridioides difficile* infection can cause severe colitis, which ultimately leads to life-threating inflammation and loss of intestinal epithelial barrier functions in a still unacceptably high fraction of patients. In this context, immune responses ultimately evoke infiltration of neutrophilic granulocytes that beside their role in bacterial clearance have been identified as major drivers of CDAC (Kelly et al., 1994; Burakoff et al., 1995; Bulusu et al., 2000). While activation of epithelial and myeloid cells can initiate a vicious cycle of intestinal inflammation, *C. difficile*-induced activation of innate lymphoid cells (ILC) can also mediate protection against the pathogen. Abt et al. (2015) have recently demonstrated that recovery from acute CDI is independent of adaptive T cell responses, while IFNγ-secreting ILC1 and to a far lesser extent IL22-producing ILC3 were clearly proven to confer protection against acute CDI in mice. IFNγ released by ILC1 and probably other innate lymphocytes may contribute to enhanced clearance of bacteria from the lamina propria, thus preventing systemic pathogen spreading. Interestingly, in addition to ILCs other innate-like T cells have been shown to play beneficial roles in CDI (Rampuria et al., 2015). Taken together, current results point toward a multifaceted role of host innate immunity in the immunopathology of CDAC and suggest a role of innate-like lymphocyte subsets in determining the outcome of CDI.

In this study, we identified human MAIT cells as a further cell type to be considered in CDI. MAIT cells have innate-like properties, including the degranulation of cytotoxic granules, and can directly respond to MR1-presented metabolites derived from bacterial riboflavin biosynthetic pathway, which was suggested to be functional in *C. difficile* strains before (Liuzzi et al., 2015; Magnúsdóttir et al., 2015). We observed earliest MAIT cell activation after 12 h with a peak response against *C. difficile* after 17 h. This delay in MAIT cell activation can be explained by the time required for MR1^+^ antigen presenting cells to process and present suitable amounts of MR1-binding ligands to MAIT cells but might be different when using living instead of fixed bacteria (Kurioka et al., 2015). After antigen recognition, MAIT cells first increased their expression of activation marker (CD69) and cytotoxic effector molecules (perforin, GzmB) followed by a predominant cytokine-dependent degranulation (Figure 3). At the same time, *C. difficile*-activated MAIT cells produced the proinflammatory cytokine IFNγ. Murine studies have uncovered IFNγ as one factor conferring protection against acute CDI (Abt et al., 2015) and our current study provides first experimental data that in addition to murine ILC1, human MAIT cells are able to mount an IFNγ response toward *C. difficile*. Abt et al. speculated that IFNγ may strengthen the immunological barrier in the intestine and thus playing a beneficial role in the control of acute CDI (Abt et al., 2015). In this scenario, MAIT cells in humans might play a protective role in CDI similar to murine ILC1, whereby their IFNγ response seemed to be mostly controlled by antigen-presentation. Based on our observation that IFNγ could not significantly be inhibited by IL-12/IL-18 blockade, we found *C. difficile*-induced IFNγ expression of primary human MAIT cells mostly MR1-dependent. This suggests the existence of a pathogen-specific mechanism in MAIT cell activation. However, IL-12/IL-18 has an impact on IFNγ expression, since IL-12/IL-18 alone primes IFNγ expression in MAIT cells and an additional MR1/TCR stimulation results in pronounced IFNγ expression in individual donors (Supplementary Figure 3). This finding is in accordance with *in vitro* studies using *E. coli*, which reported IFNγ expression by MAIT cells to co-depend on MR1/TCR- and IL-12/IL-18 signaling after 20 h culture (Ussher et al., 2014). Data from this and earlier time points indicate that *C. difficile*-induced IFNγ expression is mostly MR1-dependent, suggesting that the cell contact with antigen-presenting cells is essential for IFNγ responses in the acute phase of infection. In contrast, CD69 expression and the expression of cytotoxic perforin and GzmB require both MR1 and IL-12/IL-18 and potentially other MAIT cell activating factors or cytokines (e.g., IL-15) that can support the induction of cytotoxic effector molecules (Chiba et al., 2017).

With respect to MAIT cell activation kinetics, we observed a temporal peak of perforin and GzmB expression at 17 h post stimulation with *C. difficile*. This kinetic very much resembles the expression profile of cytolytic molecules induced in *E. coli*-stimulated MAIT cells (Kurioka et al., 2015). While the expression of effector molecules in *C. difficile*-stimulated MAIT cells indicate arming of lytic granules and the adaptation of their prototypic granzyme pattern including GzmB, GzmA and GzmK (Kurioka et al., 2015; Bulitta et al., 2018), we additionally examined potential effects on MAIT cell degranulation. Indeed, we found CD107a surface expression peaking 17 h post stimulation with *C. difficile* indicating lytic granule release. Therefore, granzymes together with perforin are likely available at elevated level both intracellularly in *C. difficile*-activated MAIT cells as well as extracellularly. However, we also observed slightly elevated levels of perforin and CD107a after 17 h in unstimulated MAIT cells, suggesting further PBMC-intrinsic processes that support lytic granule degranulation even in the absence of *C. difficile*. In our study, the pathogen-induced degranulation peak was characterized to be MR1-independent and could only be blocked by the addition of IL-12/IL-18 antibodies. It is therefore tempting to speculate that MAIT cells, once activated MR1/TCR-dependently by *C. difficile*, might be able to release lytic granules with known MAIT effector components in a cytokine-dependent manner but independent of further MR1/TCR binding.

The key question is, whether TCR engagement and the formation of immunological synapses between MAIT cells and target cells at all are dispensable. An undirected release of cytotoxic components would certainly support tissue damage and excessive inflammation ultimately leading to decreased epithelial barrier function. Furthermore, activated MAIT cells at the mucosa can produce IL-17 (Dusseaux et al., 2011; Gibbs et al., 2017) that would additionally promote neutrophil-mediated inflammatory responses (Liu et al., 2016). Together, MAIT cells, although generally able to kill infected cells, might be instrumental for *C. difficile* to overcome mucosal barriers and therefore might play a so far unrecognized detrimental role in inflammation-driven immunopathology observed in CDAC.

This hypothesis is further corroborated by our observation that hypervirulent *C. difficile* strains, despite producing only moderate levels of riboflavin during anaerobic *in vitro* culture, are superior in activating MAIT cell effector responses. However, we consider clostridial riboflavin concentrations likely not to be sufficient to predict the level of MR1-binding intermediates. The generation of MAIT cell-activating ligands depends on several parameters including the activity of *ribD* as wells as non-enzymatic reactions, whereas further processing depends on *ribE* (Corbett et al., 2014). The higher the activity of *ribE* compared to *ribD*, the higher is the conversion of MR1-binding ligand (5-AR-U) into riboflavin. In non-enzymatic reactions with glyoxal and methylglyoxal, the MR1-binding ligand (5-AR-U) is converted into 5-OP-RU and 5-OE-RU, which have the highest MAIT-activating potency (Schmaler et al., 2018). Up to now there is no experimental data determining the amount of MR1-binding metabolites (5-A-RU, 5-OP-RU, and 5-OE-RU) in *C. difficile*, because isolation and analysis of intermediates is technically difficult due to intermediates’ short half-life and instability when not bound to MR1 (Corbett et al., 2014; Schmaler et al., 2018). Assuming a relatively high activity of *ribD* could result in the accumulation of MAIT cell-activating metabolites in *C. difficile*, this could be a possible explanation for superior activation of MAIT cells by hypervirulent *C. difficile* isolates despite only moderate riboflavin concentrations in the cultures. In general, it is imperative to understand the interdependency between MR1-antigen generation and riboflavin metabolism better. Preliminary experiments with a *rib gene* deletion mutant (based on *C. difficile* strain 630Δerm) indicate that riboflavin synthesis is not essential for *C. difficile* growth (data not shown). The use of such mutant strain will facilitate perspective functional and *in vivo* studies.

One might also speculate that the hypervirulent strains induce, due to their toxins, a largely altered cytokine pattern in epithelial and myeloid cells, which in turn would affect TCR-independent MAIT cell activation mediated by cytokines (Ussher et al., 2014; Slichter et al., 2016). We here showed that proinflammatory cytokines including IL-12 and IL-18 derived from immune cells in PBMC cultures have an impact on inducing potent cytotoxic response by MAIT cells (Figure 2). Moreover, we could show that the hypervirulent strains induce a higher IL-12 secretion by immune cells in the PBMC culture than non-hypervirulent strains. (Kurioka et al., 2015) reported granzyme B and perforin as IL-12- but not IL-18-dependent. This could also be a possible explanation for superior cytotoxic response of MAIT cells by hypervirulent *C. difficile* isolates. However, further experiments are required to dissect IL-12 and IL-18 specific effects on *C. difficile*-stimulated MAIT cells. Moreover, MR1-dependent MAIT responses characterized in this study might vary from MAIT cell responses at the mucosa where numerous cell types express MR1 and potentially contribute to the outcome.

In conclusion, this study provides the first characterization of a *C. difficile*-induced effector phenotype in human MAIT cells, which includes cytotoxic responses that might be instrumental for *C. difficile* to overcome epithelial barriers at the intestinal mucosa. Indeed, we identified superior MAIT cell activation by hypervirulent *C. difficile* isolates suggesting MAIT cells as novel decision makers for disease severity in CDAC.

## Ethics Statement

This study was carried out in accordance with the recommendations of the Medical Association of Lower Saxony with written informed consent from all subjects. All subjects gave written informed consent in accordance with the Declaration of Helsinki. The protocol was approved by the Institutional Review Board of the Hanover Medical School.

## Author Contributions

LJ and DB conceived and designed the research. IB and BB designed and performed the experiments and analyzed the data. JH and MN-S provided bacteria and performed the riboflavin analyses. FK designed and performed the statistical analyses. AM-M and DJ have performed the riboflavin pathway analysis. IB, LJ, and DB wrote the manuscript.

## Conflict of Interest Statement

The authors declare that the research was conducted in the absence of any commercial or financial relationships that could be construed as a potential conflict of interest.
