# Supplementary material for: Clostridioides difficile Activates Human Mucosal-Associated Invariant T Cells
Source: Front Microbiol. 2018 Oct 25;9:2532. doi: 10.3389/fmicb.2018.02532 (PMC6209678; doi:10.3389/fmicb.2018.02532)
Supplement: Supplementary file 1 [file Data_Sheet_1.docx]

Supplementary Material

***Clostridioides difficile* activates human mucosal-associated invariant T cells**

Isabel Bernal, Julia Danielle Hofmann, Björn Bulitta, Frank Klawonn, Annika-Marisa Michel, Dieter Jahn, Meina Neumann-Schaal, Dunja Bruder^*^, Lothar Jänsch^*^

^*^ Co-corresponding authors: Dunja Bruder: [dunja.bruder@med.ovgu.de](mailto:dunja.bruder@med.ovgu.de) Lothar Jänsch: [lothar.jaensch@helmholtz-hzi.de](mailto:lothar.jaensch@helmholtz-hzi.de)

**Supplementary figures**


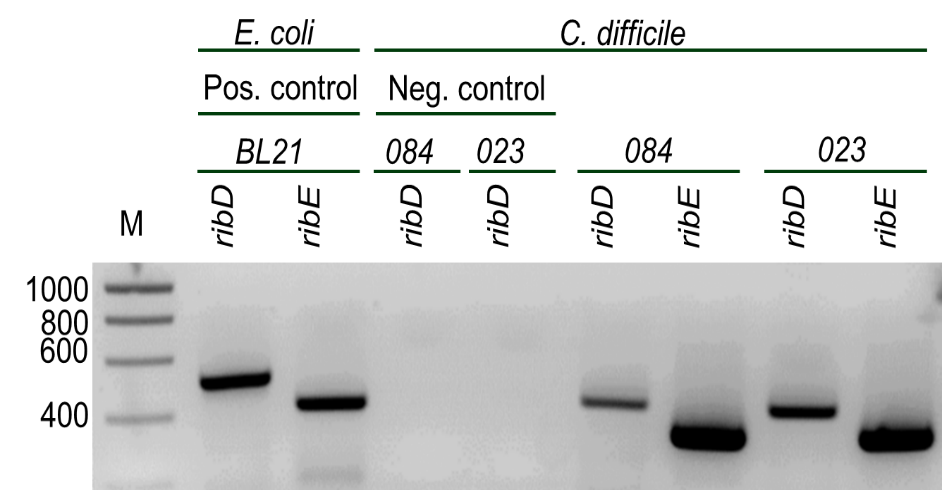


Supplementary Fig. 1: Functional riboflavin biosynthesis pathway in *C. difficile*. *C. difficile* strains of ribotype RT084 and RT023 were cultured overnight in riboflavin-free CDMM medium. Total RNA was isolated followed by RT-PCR analysis of *ribD* and *ribE* gene expression. RNA isolated from *E. coli* (BL21) cultured in LB Medium overnight served as positive control, RT-PCR using a template without prior reverse transcription served as negative control. Smart DNA ladder was used as standard molecular-weight maker (M). Expected DNA bands for *E. coli* shown: *ribD* (500 bp) *ribE* (440 bp). Expected DNA bands for *C. difficile* shown: *ribD* (450 bp) and *ribE* (350 bp). Representative results from one of two independent experiments are shown.

**
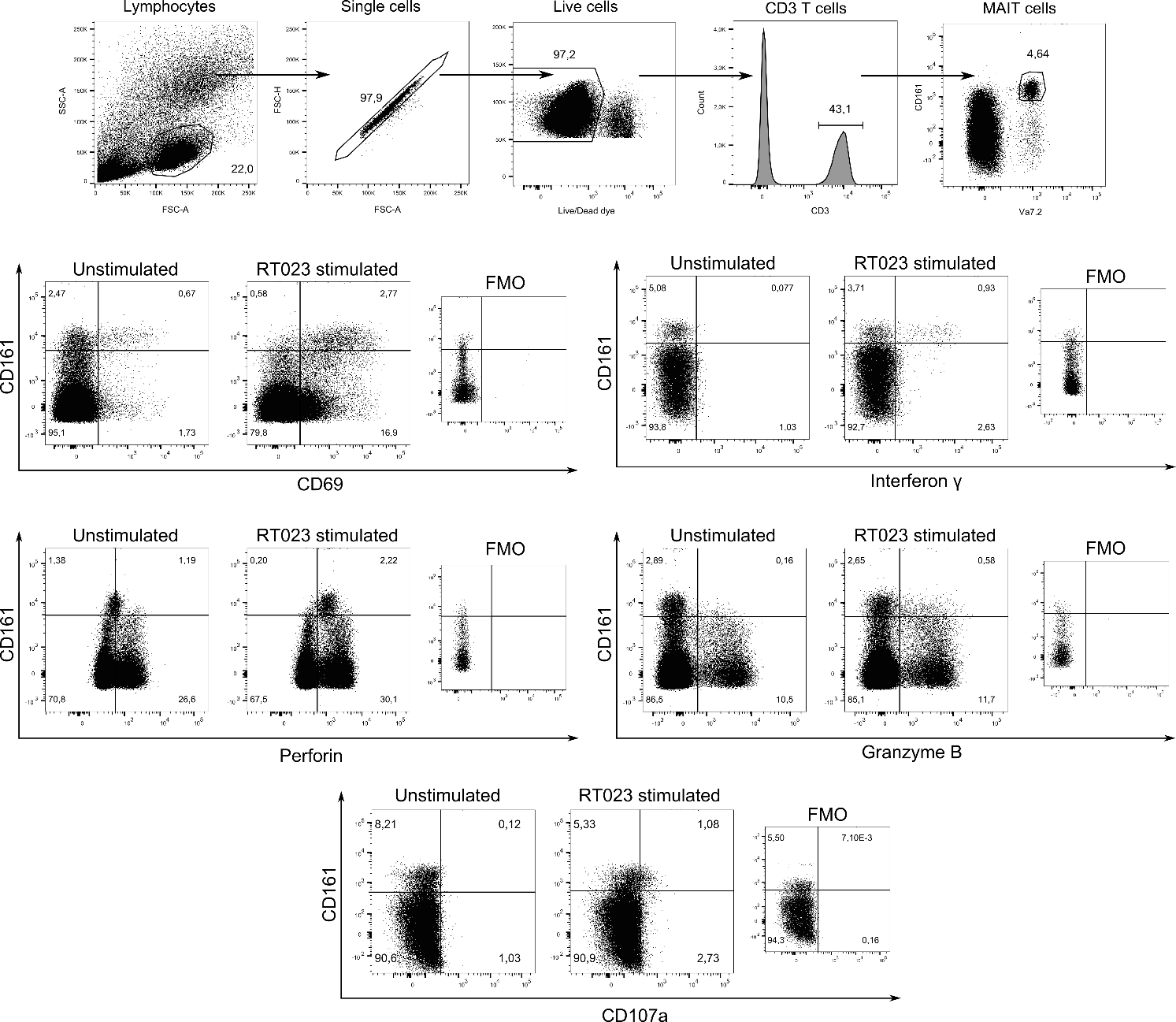
Supplementary Fig. 2: Gating strategy for human mucosa-associated invariant T (MAIT) cells.** PBMCs were isolated from human blood; live cells were discriminated with Live/Dead dye. Cells were stained with antibodies specific for CD3, Va7.2, CD161, CD69, Interferon γ, perforin, granzyme B, and CD107a followed by flow cytometric analysis. Fluorescence minus one (FMO) staining controls are shown.


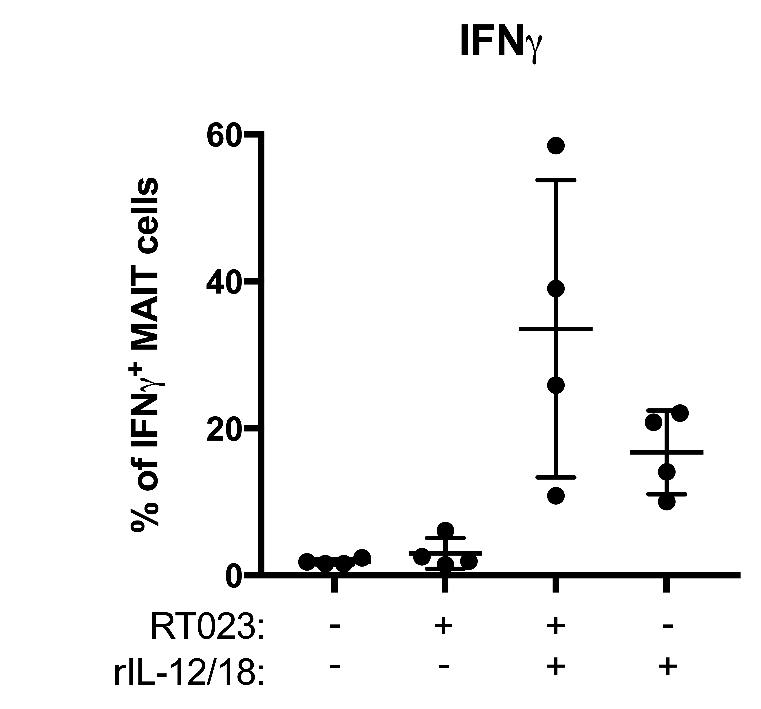


Supplementary Fig. 3: Effects of IL-12 and IL-18 on IFNγ response of primary human MAIT cells following stimulation with *Clostridioides difficile* (ribotype RT023). PBMCs were isolated from healthy donors (n=4) and stimulated with *C. difficile* clinical isolate with ribotype RT023 at MOI 1 and/or with human recombinant IL-12(p70) (BioLegend) and IL-18 (R&D systems) at 100 ng/ml for 20 h followed by flow cytometric analyses of IFNγ response. Mean percentages ± SD are shown. Cells were gated on CD161^++^Va7.2^+^CD3^+^ T cells (MAIT cells).
